# Development and psychometric properties of Midwives' Knowledge and Practice Scale on Respectful Maternity Care (MKP-RMC)

**DOI:** 10.1371/journal.pone.0241219

**Published:** 2020-11-03

**Authors:** Maryam Moridi, Farzaneh Pazandeh, Sepideh Hajian, Barbara Potrata

**Affiliations:** 1 Department of Midwifery and Reproductive Health, Student Research Committee, School of Nursing and Midwifery, Shahid Beheshti University of Medical Sciences, Tehran, Iran; 2 School of Health Sciences, University of Nottingham, Nottingham, United Kingdom; 3 Midwifery and Reproductive Health Research Center, School of Nursing and Midwifery, Shahid Beheshti University of Medical Sciences, Tehran, Iran; 4 Independent Scholar, Rotterdam, The Netherlands; University of Oxford, UNITED KINGDOM

## Abstract

**Objective:**

To develop a scale for evaluating knowledge and practice of midwives on Respectful Maternity Care (RMC).

**Methods:**

An exploratory sequential mixed method study was conducted from January 2018 to July 2019 in two non-teaching public hospitals in Tehran, Iran. In the first part of the study, a literature review and qualitative study were carried out in order to develop the preliminary item pool. Then face, content and construct validity and reliability (internal consistency and test-retest) were assessed.

**Results:**

The MKP-RMC scale has 23-item in knowledge and 23-item in practice section that loaded in three factors: Giving emotional support, providing safe care and preventing mistreatment. Exploratory factor analysis accounted for 43.47% and 58.62% of observed variance in knowledge and practice sections, respectively. The internal consistency and internal correlation coefficient of both section of MKP-RMC indicated acceptable reliability.

**Conclusion:**

The MKP-RMC is a valid and reliable tool for measuring midwives' knowledge and practice of respectful care during labor and childbirth. The MKP-RMC could be used in maternity services to evaluate and improve quality of childbirth care through development of educational interventions for effective behavioral change. Confirmation of validity and reliability of translated version of the scale in other maternity care providers and different contexts is recommended.

## 1. Introduction

The efforts for improving maternal health are changing from an emphasis on enhancing maternity service use to improving quality of care worldwide [1]. There is a consensus that promoting Respectful Maternal Care (RMC) is an important approach to improve the quality of maternity care and service utilization [2]. RMC is based on essential rights of laboring women, babies and their families. It emphasis on evidence-based childbirth care and also refers to care delivered to all women in a way that preserves their dignity, privacy and confidentiality, supports informed choice and continuous support for labouring women, ensures preventing disrespect and abuse during labour and childbirth [3]. This is important because reports from low, middle and high-income countries demonstrate that women’s dignity during childbirth is not always adequately protected [2, 4].

The White Ribbon Alliance (WRA) (2011) is a global movement which argues the need for complex interventions for effective implementation of the RMC. The main drivers of mistreatment in maternity services have been identified as structural factors of healthcare systems and they include poor policy, inadequate provider training, deficient supervision and weak midwifery management [4, 5]. As behavioral change of providers has been recognized as the main barrier to better care [6], improving providers’ awareness of principles of RMC has become an essential component for implementation of RMC and improving childbirth experience worldwide [7, 8]. The midwives have a key role in providing maternity care [9, 10] and their knowledge, competence, communication skills, professional and personal development are fundamental for implementing quality care during birth [11, 12]. Several studies have explored providers’ views on RMC [7, 13, 14], but there is no valid and reliable scale to assess their awareness and performance of RMC [8]. There is a need for specific and practical tool for evaluating knowledge and performance of midwives to improve maternity and childbirth care.

## 2. Aim of the study

This study therefore aimed to develop a scale for evaluating knowledge and practice of midwives on RMC.

## 3. Materials and methods

### 3.1. Study design

This study was conducted from January 2018 to July 2019 in Iranian capital city Tehran. The study used exploratory sequential mixed methods approach as it provides an original approach for designing a valid and reliable context-based scale. First a qualitative research was done, and the results of that were used to design the scale, then the psychometric properties of scale were investigated through a quantitative study.

### 3.2. Data sampling

The data was obtained from a representative sample of the Iranian midwives regarding age, educational level and years of work experience. The inclusion criteria were having at least bachelor’s degree in midwifery and one year or more work experience in labor and childbirth unit. Cluster sampling was used for selection of 15 districts randomly from all 22 districts of Tehran. Then, we randomly selected one hospital in each district and midwives were recruited by census method in each hospital.

### 3.3. Stages of development

The development and validation of items related to knowledge and practice of midwives were undertaken in five phases: 1. Item generation, 2. Content and face validity, 3. A pilot study for item analysis, 4. Construct validation and 5. Reliability assessment.

### 3.4. Phase 1: Item generation of MKP-RMC by using literature review and a qualitative study

The item identification, selection and development started with a literature review and search for articles and scales related to the measurement of RMC. Five databases (PubMed, CINAHL, Embase, MEDLINE and Psych Info) were searched from 2000 to 2018, looking for articles in English and Persian language. Following MESH terms were used: knowledge, attitude, practice, behavior, personal narrative, experience, perception, satisfaction, health care provider, midwife, nurse-midwife care, respect, dignity, humanization, human right, abuse, disrespect, childbirth, facility birth, hospital birth, parturition, maternity services. The identified papers described providers and childbearing women’s experiences of disrespect or respectful care during labor and childbirth. Reference lists of these papers were also examined. Literature review was followed by a qualitative study, the details of which are described elsewhere [15].

### 3.5. Phase 2: Face and content validation of MKP-RMC

#### 3.5.1. Face validity

The quantitative and qualitative methods were applied to establish face validity. In the quantitative part, 20 experts in midwifery and reproductive health were asked to evaluate the questionnaire and score the importance of each item in order to calculate ‘Item Impact Score’. The impact score of 1.5 or above was considered satisfactory as recommended by McDowell (2006) [16]. To determine qualitative face validity, the same midwives were asked about the ‘relevancy’, ‘ambiguity’, and ‘difficulty’ of the items; and some minor changes were made to the preliminary questionnaire.

#### 3.5.2. Content validity

The expert panel consisted of 15 specialists in midwifery, reproductive health, nursing and medical ethics. Qualitative content validity was determined based on ‘grammar’, ‘wording’, ‘item allocation’, and ‘scaling’ indices. All items were checked and the expert panel’s recommendations were inserted into the questionnaire. Content Validity Ratio (CVR) and Content Validity Index (CVI) were calculated in order to perform quantitative content validity. According to Lawshe’s table (1975), only items with CVR score of 0.49 or above were selected [17]. Based on Waltz and Bausell recommendation, a CVI score of 0.80 or above was considered satisfactory [18].

### 3.6. Phase 3: A pilot study for item analysis of MKP-RMC

Item analysis was done by calculating discrimination index which assessed the item-total correlation using Spearman's correlation coefficient in a sample of 50 midwives. Item discrimination was considered as good if the index was above 0.30 [19]. Such items were kept in the scale.

### 3.7. Phase 4: Construct validation of MKP-RMC by using factor analysis

For EFA, a sample of 250 midwives completed the questionnaire and its factor structure was extracted using the principal axis factoring for knowledge scale and maximum likelihood for practice scale with promax rotation. The Kaiser-Meyer-Olkin (KMO) and Bartlett’s Test of Sphericity were used to assess the appropriateness of the sample for the factor analysis. The number of factors was fixed at three, based on the scree plot suggestion of the optimum number of factors. Factor loadings equal or greater than 0.4 were considered appropriate [20, 21].

### 3.8. Phase 5: Reliability assessment of MKP-RMC

Internal consistency was evaluated by Kuder Richardson 20 (KR20) (for binary scale) and Cronbach’s α (for 5-point scale) coefficients. KR20 and Cronbach’s α coefficient of 0.7 or above was considered satisfactory [18]. In addition, a sub-sample of midwives (n = 30) completed the questionnaire twice with a two week interval in order to examine the stability of the scale by calculating Intra-class Correlation Coefficient (ICC) where the ICC of 0.8 or above was considered acceptable [22].

### 3.9. Statistical analysis

All statistical analysis for item analysis, construct validation and reliability assessment were performed using the SPSS version 21.0 at the p = 0.05 significance level.

### 3.10. Scoring

All items of the knowledge scale, were dichotomized with 1 representing endorsement of the item or agreement and 0 representing non-endorsement or disagreement with the item. The practice scale consisted of items that were originally assessed using a five-point Likert scale, and categorized as always, often, sometimes, rarely and never (scored 5 to 1). In both sections of the scale, 21, 22 and 23 items have scored reversely. A composite score was then created by summing all the individual items within each scale. The highest scores of knowledge and practice scales are 23 and 115 and the lowest scores are 0 and 23 respectively.

#### 3.10.1. Ethics

This study was approved by the ethics committee of Shahid Beheshti University of Medical sciences approved the study (IRB code = 1396.810) and the authorities in the selected hospitals. The potential participants were given a detailed description of the study and were assured confidentiality. The participants were informed about that they would be free to drop out of the interview at any time or not to complete the questionnaire without negative consequences.

## 4.Results

### 4.1. Item generation

In the present study, 110 items were extracted from literature review and qualitative study, organised as three thematic domains and seven sub-themes. Then, the research team asessed and compared these items to generate the first draft of MKP-RMC with 74 items (37 items in knowledge and 37 items in practice sections). This was followed with the evaluation of face and content validation.

### 4.2. Samples in quantitative phase of the study

The samples were 250 midwives with the average age of 33.33 ± 8.75 (ranged from 21 to 58 years) and average working experiences in labor and birth units 8.50±7.67 (ranged from 1 to 29 years). The majority of participants were married (60%), had bachelor degree in midwifery (80.4%), and permanent job (56%). Majority of midwives had childbirth experience (62.4%) and less than two children (97.2%) (Table 1).

**Table 1:** The demographic characteristics of midwives.

Characteristics	Data for EFA (n = 250) n (%)
Age (year)	
>25	49 (19.6)
25–35	110 (44)
35–45	61 (24.4)
<45	30 (12)
Marital status	
Single	100 (40)
Married	150 (60)
Educational level	
Associate Degree	
Bachelor	8 (3.2)
MSc	201 (80.4)
	41 (16.4)
Work experience in birth unit (years)	
>5	124 (49.6)
5–10	51 (20.4)
10–15	30 (12)
15–20	18 (7.2)
20–25	16 (6.4)
< 25	11 (4.4)
Having childbirth	94 (37.6)
Yes	156 (62.4)
No	
Numbers of children	
0	155 (62)
1	41 (16.4)
2	47 (18.8)
<2	7 (2.8)
Employment status	
Permanent	140 (56)
Temporary	110 (44)

#### 4.2.1. Face and content validity

First, the face validity of the scale was assessed in terms of the importance of each item. Following assessment of qualitative face validity, 22 and 21 items in knowledge and practice sections were modified respectively. Then the quantitative face validity was assessed and impact score of all items were more than 1.5 were kept except for 3 and 9 items in the knowledge and practice sections, respectively.

The MKP-RMC scale (34 items in knowledge and 28 items in practice sections) was then assessed for the content validity and CVR and CVI of each item were calculated. The items with the CVR scoring 0.49 or higher as well as CVI scoring 0.80 or higher were kept. As a result, 6 items in knowledge and 1 item in practice sections were removed. Several ambiguous items were modified for better comprehension. At the end of this stage, the MKP-RMC scale comprised of 28 knowledge and 27 practice items.

#### 4.2.2. Item analysis

One item in each scale was removed as their correlation coefficients were less than 0.3.

#### 4.2.3. Exploratory factor analysis

The Kaiser-Meyer-Olkin (KMO) index for knowledge and practice sections were 0.77 and 0.92 respectively, and the Bartlett’s tests of sphericity were significant (2.76 and 4.35 respectively, P<0.001). The initial analysis indicated an 8-factor structure for the knowledge and 4-factor structure for practice sections. The eigenvalues of knowledge and practice sections were greater than one that accounted for 67.52% and 63.07% of the observed variance respectively. Factor loading pattern in scree plot figures of knowledge and practice sections indicated that after three factors, the diagrams' slope plateaued. Based on the low loading of some factors in scree plot diagrams, the number of factors was fixed in three in both sections of MKP-RMC scale. The factor loading was set at a minimum of 0.4. Finally, 23 items in each section were loaded in three factors that explained 43.47% and 58.62% of the variance respectively. The three factors of each section were represented the following dimensions: giving emotional support (12 and 11 items), providing safe care (8 and 9 items) and preventing mistreatment (3 and 3 items), (Tables 2 and 3(.

**Table 2:** The exploratory factor analysis using the principal axis with promax rotation of knowledge sections.

Items	Giving emotional support	Providing safe care	Preventing mistreatment
1. Warm welcoming in entering to labor unit	0.446		
2. Showing around maternity labor unit's environment	0.684		
3. Establishing friendly communication	0.759		
4. Encouraging and giving calming touch	0.602		
5. Calling laboring woman’s name as she desires	0.567		
6. Providing accurate and clear information about progress of labor, received care and interventions	0.469		
7. Providing friendly environment to ask questions	0.490		
8. Providing comfortable and calming environment	0.497		
9. Freedom in choosing birthing position	0.514		
10. Having companion of choice upon request	0.490		
11. Respecting laboring woman’s and her companions' beliefs and culture	0.462		
12. Providing appropriate environment for companions	0.488		
13. Continuous or timely presence beside		0.432	
14. Keeping medical records and the results of tests and consultations confidential		0.518	
15. Obtaining informed consent before performing any care and interventions		0.473	
16. Providing equal care to all laboring woman regardless of their socio-economic status, ethnicity, etc.		0.535	
17. Providing evidence-based and up-to-date childbirth care		0.416	
18. Providing pain relief		0.472	
19. Paying attention to safety in providing care and interventions		0.788	
20. Providing accurate information about progress of labor to companions		0.587	
21. Attendance of unnecessary person during performing procedure			0.765
22. Physical violence in the case of non-cooperation			0.755
23. Shouting at the laboring woman in case of non-cooperation			0.729
Eigenvalues	5.74	5.68	2.85
% Explained variance	26.96	9.66	6.83
% Cumulative variance	26.96	36.63	43.47

**Table 3:** The exploratory factor analysis using the maximum likelihood with promax rotation of practice scale.

Items	Giving emotional support	Providing safe care	Preventing mistreatment
1. I welcome laboring woman warmly.	0.660		
2. I introduce myself to the laboring woman.	0.510		
3. I show the laboring woman around the labor unit.	0.733		
4. I establish friendly and appropriate relationship with the laboring woman.	0.864		
5. I support laboring woman by encouraging and calming touch.	0.943		
6. I use the name preferred by a laboring woman.	0.870		
7. I am continuously or timely available beside.	0.803		
8. I provide laboring woman with correct and clear information about the care, interventions and progress of labor.	0.714		
9. I build friendly relationship in a way that she feels comfortable to ask her questions.	0.748		
10. I provide a comfortable environment for laboring woman.	0.683		
11. I support laboring woman to be in her desired birthing position.	0.737		
12. I keep medical records and the results of examinations and consultations confidential.		0.674	
13. I cover the laboring woman’s body during examinations, using sheets.		0.761	
14. I perform all interventions with laboring woman’s informed consent.		0.725	
15. I provide equal care to all women, regardless of their socio-economic status, ethnicity, etc.		0.891	
16. I support laboring woman to take care of herself and her baby.		0.703	
17. I provide evidence-based and up-to-date childbirth care.		0.724	
18. I pay attention to laboring woman’s safety in providing care and interventions.		0.781	
19. I respect beliefs and culture of laboring woman and her companions.		0.470	
20. I provide companions with accurate and clear information about progress of labor.		0.445	
21. I do not allow the laboring woman to have companion inside the labor unit.			0.474
22. I may beat the laboring woman if she does not cooperate.			0.983
23. I may shout at laboring if she does not cooperate.			0.903
Eigen values	9.83	9.03	2.60
% Explained variance	41.97	10.40	6.28
% Cumulative variance	41.97	52.37	58.66

#### 4.2.4. Reliability

The knowledge and practice sections of MKP-RMC scale had good internal consistency (0.72 and 0.95, respectively). The intra-class correlation coefficients in knowledge and practice sections were 0.92 and 0.79, respectively indicating an appropriate stability of the scale (Table 4).

**Table 4:** Reliability (internal consistency and internal correlation coefficient).

Factors	Kuder-Richardson 20	Cronbach’s alpha	ICC	
	Knowledge	Practice	Knowledge	Practice
**Giving emotional support**	0.89	0.9	0.79	0.89
**Providing safe care**	0.72	0.81	0.82	0.83
**Preventing mistreatment**	0.77	0.85	0.81	0.88
**Whole Questionnaire (Is this total score?)**	0.91	0.95	0.90	0.92

## 5. Discussion

This study developed and assessed the psychometric properties of Midwives' Knowledge and Practice on Respectful Maternity Care (MKP-RMC) scale using an exploratory sequential mixed method approach. The psychometric assessment demonstrated that the MKP-RMC is a valid and reliable scale to evaluate providers’ knowledge and practice of RMC. The face, content and construct validity of scale and the reliability (internal consistency and internal correlation coefficient) were acceptable.

The MKP-RMC consisted of 46 items in both knowledge and practice sections (23 items in each) and organized into three domains, namely giving emotional support, providing safe care and preventing mistreatment. The initial questionnaire was developed based on data obtained from extensive review of the existing literature on laboring women’s experiences of RMC and a qualitative interview study with midwives on their perspectives of RMC. This scale includes items to assess significant factors which are in line with the main concepts of the Universal Rights of Childbearing Women charter [6].

To the best of our knowledge, measuring knowledge and practice of midwives on RMC is relatively a new area, not only in Iran but also elsewhere. Ndwiga et al. in Tanzania, (2017) used an extensive and general tool to assess the knowledge, attitudes and behavior of providers during labor and birth in Tanzania [8]. Their scale consisted of 11 domains and 115 items. The domains include individual, interpersonal, managerial and structural factors affecting RMC [8]. The domains of MKP-RMC scale are relatively in accordance with the scales which have developed to assess women’s experience of RMC [23–25] Recently, Ayoubi et al., (2020) developed the Women's Perception of Respectful Maternity Care scale (WP-RMC) in Iran with three domains, including providing comfort, participatory care and mistreatment [23]. Sheferaw et al., (2016) in Canada also developed a scale with four domains which include friendly, abuse-free, discrimination-free and timely care [24]. Vedam et al., (2017) introduced MOR index in Ethiopia with domains on autonomy and comfort, modified behavior and perceptions of discrimination [25].

The first domain of our scale is ‘giving emotional support‘ which includes the greatest number of items in MKP-RMC scale. One of the items of this domain is ‘establishing friendly communication’ that has the highest loading factor in both knowledge and practice sections. The items of this domain were similar to the items in the ‘friendly care’ domain in 15-item scale of Sheferaw et al. (2016) [23, 25], and ‘sense of autonomy and comfort’ domain in MOR index of Vedam et al., 2017 [24]. Other studies have also confirmed that pregnant women value emotional support more than other aspects of maternity care [26, 27]. Previous studies showed that effective communication with laboring women and their families is a cornerstone for providing quality health care. Therefore, ineffective communication can lead to anxiety, vulnerability, and powerlessness [7, 28].

The second domain in MKP-RMC scale is ‘providing safe care’. This domain indicates the necessity of providing confidentiality, privacy, and respect for women's religious beliefs and culture. It also stresses the importance of providing evidence-based care and information about progress of labor, received care and interventions during childbirth. The items of this domain are in accordance with the laboring women's rights reported by WRA [6]. ‘Providing equal care for all women’ has the highest loading factor in our scale. The importance of these concepts is supported by other studies that show the key role of midwives respecting women's culture, values and beliefs. [29, 30]. In addition, this domain and its items are relatively similar to other related scales that have been developed to evaluate women’s experiences of RMC [23–25].

The third domain of the questionnaire ‘preventing mistreatment’, refers to the context of maintaining dignity of women during labor and childbirth. The items with the highest loading factor were “attendance of unnecessary person during performing procedure” and "I may beat the laboring woman if she does not cooperate" in knowledge and practice scale respectively. This domain is about violation of the women's rights, and it is close to ‘avoiding disrespect’ in the RMC scale of Sheferaw et al (2016) [23]. Additionally, ‘Physical violence in the case of non-cooperation’ and ‘Shouting at clients in the cases of non-cooperation’ items in the MKP-RMC scale are equal to ‘My caregivers bet me’ and ‘My caregivers shouted at me if I did not follow their instructions’ in WP-RMC scale of Ayoubi et al (2020) [25].

### 5.1. Strengths and limitations

The MKP-RMC is a multi-demensional scale that has been developed using comprehensive literature review and perceptions of midwives and postpartum women via in-depth interviews from different regions of Iran, and asssessed using robust statistical methods.

There are some limitation to note. The binary response format (yes, no) in the knowledge section of MKP-RMC scale resulted in low response variance. A five-point Likert format may be more effective at capturing awarness about RMC. Furthermore, the practice scale is a self-reported construct and might be a limitation of this scale. However, self-report method for measuring behavior is accepted method in scale development [31] and its effectiveness was confirmed in several studies [32, 33].

## 6. Conclusion

The MKP-RMC is a valid and reliable tool that could be used for assessing knowledge and practice of providers in the maternity services. This tool could be used in the settings that aimed to assess and improve quality of care and contribute to development of educational interventions for behavioral change. This scale is also recommended for assessment of knowledge and practice of different providers of maternity care in other contexts. Assessment of psychometric properties of the MKP-RMC in different settings would contribute to a stronger confirmation of psychometric robustness of this scale.

## Supporting information

S1 File (DOC)

S2 File (DOC)

## Abbreviations

MKP-RMC: Midwives Knowledge and Practice scale on Respectful Maternity Care
RMC: Respectful Maternity Care
WRA: White Ribbon Alliance
WHO: World Health Organization
