# Supplementary material for: Development and psychometric properties of Midwives' Knowledge and Practice Scale on Respectful Maternity Care (MKP-RMC)
Source: PLoS One. 2020 Nov 3;15(11):e0241219. doi: 10.1371/journal.pone.0241219 (PMC7608882; doi:10.1371/journal.pone.0241219)
Supplement: S1 File — (DOC) [file pone.0241219.s001.doc]

پرسشنامه سنجش آگاهی و عملکرد مامای در زمینه مراقبت مامایی کرامت محور (MKP-RMC)

| **بخش آگاهی** | | | | |
| --- | --- | --- | --- | --- |
| **شماره** | **گویه ها** | **بله** | **خیر** | **نمی دانم** |
| 1 | خوشامدگویی گرم در بدو ورود به لیبر |  |  |  |
| 2 | آشنا نمودن با محیط لیبر |  |  |  |
| 3 | برقراری ارتباط دوستانه |  |  |  |
| 4 | تشویق و نوازش آرامش بخش |  |  |  |
| 5 | صدا کردن اسم بر اساس ترجیح زائو |  |  |  |
| 6 | فراهم نمودن اطلاعات صحیح و قابل فهم در مورد پیشرفت زایمان، مراقبت ها و مداخلات |  |  |  |
| 7 | فراهم نمودن محیط صمیمی جهت پرسیدن سوالات |  |  |  |
| 8 | فراهم نمودن محیطی راحت و آرام |  |  |  |
| 9 | آزادی در انتخاب وضعیت مناسب در طی لیبر و زایمان |  |  |  |
| 10 | امکان حضور همراه منتخب زائو |  |  |  |
| 11 | احترام به فرهنگ و اعتقادات زائو و همراهان وی |  |  |  |
| 12 | فراهم نمودن محیطی مناسب برای همراهان |  |  |  |
| 13 | حضور مداوم یا به موقع بر بالین |  |  |  |
| 14 | محرمانه ماندن مشخصات پرونده پزشکی، نتایج معاینات و مشاوره ‎ها |  |  |  |
| 15 | اخذ رضایت آگاهانه قبل از انجام هرگونه مراقبت و مداخله |  |  |  |
| 16 | فراهم نمودن مراقبت مامایی برابر برای همه زنان زائو بدون توجه به وضعیت ظاهر و شرایط اجتماعی-اقتصادی، قومیت و .... |  |  |  |
| 17 | ارائه مراقبت مبتنی بر شواهد و دانش روز |  |  |  |
| 18 | فراهم نمودن روش های کاهش درد |  |  |  |
| 19 | توجه به ایمنی زائو در انجام مراقبت ها و مداخلات |  |  |  |
| 20 | فراهم نمودن اطلاعات دقیق به همراه (همراهان) در مورد پیشرفت زایمان |  |  |  |
| 21 | حضور افراد غیرضروری در هنگام اجرای مراقبت |  |  |  |
| 22 | خشونت جسمی در صورت عدم همکاری زائو |  |  |  |
| 23 | فریاد زدن در صورت عدم همکاری زائو |  |  |  |

| **بخش عملکرد** | | | | | | |
| --- | --- | --- | --- | --- | --- | --- |
| **شماره** | **در هنگام ارائه مراقبت:** | **همیشه** | **بیشتر اوقات** | **گاهی اوقات** | **به ندرت** | **هیچ وقت** |
| 1 | در بدو ورود خوش آمد گویی گرمی به عمل می آورم. |  |  |  |  |  |
| 2 | خود را به زائو معرفی می کنم. |  |  |  |  |  |
| 3 | زائو را با محیط بخش آشنا می کنم. |  |  |  |  |  |
| 4 | ارتباط مناسب و دوستانه ای با زائو برقرار میکنم. |  |  |  |  |  |
| 5 | با تشویق و نوازش، زائو را حمایت میکنم. |  |  |  |  |  |
| 6 | نام زائو بر اساس ترجیح وی صدا می کنم. |  |  |  |  |  |
| 7 | به طور مداوم و یا به موقع بر بالین زائو حاضر می شوم. |  |  |  |  |  |
| 8 | به زائو اطلاعات صحیح و قابل فهمی در مورد پیشرفت زایمان، مراقبت ها و مداخلات ارائه می دهم. |  |  |  |  |  |
| 9 | با زائو ارتباط صمیمانه ای برقرار می کنم که بتواند به راحتی سوالاتش را بپرسد. |  |  |  |  |  |
| 10 | محیطی آرام برای زائو فراهم می کنم. |  |  |  |  |  |
| 11 | به زائو اجازه می دهم آزادانه در وضعیت دلخواه خود قرار گیرد. |  |  |  |  |  |
| 12 | مشخصات پرونده پزشکی، نتایج معاینات و مشاوره ‎ها را محرمانه نگه می دارم. |  |  |  |  |  |
| 13 | بدن زائو را در هنگام معاینه با استفاده از ملحفه می پوشانم. |  |  |  |  |  |
| 14 | هرگونه مراقبت برای زائو را با رضایت آگاهانه وی انجام می دهم. |  |  |  |  |  |
| 15 | برای همه زنان صرف نظر از وضعیت ظاهر و شرایط اجتماعی-اقتصادی، قومیت و .... خدمات مامایی برابر ارائه می دهم. |  |  |  |  |  |
| 16 | زائو را برای انجام مراقبت از خود و نوزادش حمایت میکنم. |  |  |  |  |  |
| 17 | مراقبت مامایی مبتنی بر شواهد و دانش روز ارائه می دهم. |  |  |  |  |  |
| 18 | هنگام ارائه مراقبت ها و مداخلات به ایمنی زائو توجه می کنم. |  |  |  |  |  |
| 19 | به فرهنگ و اعتقادات زائو و همراهان وی احترام می گذارم. |  |  |  |  |  |
| 20 | اطلاعات قابل فهم و دقیق به همراهان در مورد پیشرفت زایمان ارائه می دهم. |  |  |  |  |  |
| 21 | به زائو اجازه داشتن همراه در داخل بخش لیبر را نمی دهم. |  |  |  |  |  |
| 22 | در صورت عدم همکاری، ممکن است زائو را بزنم. |  |  |  |  |  |
| 23 | در صورت عدم همکاری زائو، ممکن است بر سر زائو داد بزنم. |  |  |  |  |  |
