# Supplementary material for: Development and psychometric properties of Midwives' Knowledge and Practice Scale on Respectful Maternity Care (MKP-RMC)
Source: PLoS One. 2020 Nov 3;15(11):e0241219. doi: 10.1371/journal.pone.0241219 (PMC7608882; doi:10.1371/journal.pone.0241219)
Supplement: S2 File — (DOC) [file pone.0241219.s002.doc]

**Midwives' Knowledge and Practice on Respectful Maternity Care questionnaire (MKP-RMC)**

| **Knowledge section** | | | |
| --- | --- | --- | --- |
| **Items** | **Yes** | **No** | **I do not know** |
| 1. Warm welcoming in entering to labor unit |  |  |  |
| 2. Showing around maternity labor unit's environment |  |  |  |
| 3. Establishing friendly communication |  |  |  |
| 4. Encouraging and giving calming touch |  |  |  |
| 5. Calling laboring woman’s name as she desires |  |  |  |
| 6.Providing accurate and clear information about progress of labor, received care and interventions |  |  |  |
| 7. Providing friendly environment to ask questions |  |  |  |
| 8. Providing comfortable and calming environment |  |  |  |
| 9. Freedom in choosing birthing position |  |  |  |
| 10. Having companion of choice upon request |  |  |  |
| 11. Respecting laboring woman’s and her companions' beliefs and culture |  |  |  |
| 12. Providing appropriate environment for companions |  |  |  |
| 13. Continuous or timely presence beside |  |  |  |
| 14. Keeping medical records and the results of tests and consultations confidential |  |  |  |
| 15. Obtaining informed consent before performing any care and interventions |  |  |  |
| 16. Providing equal care to all laboring woman regardless of their socio-economic status, ethnicity, etc |  |  |  |
| 17. Providing evidence-based and up-to-date childbirth care |  |  |  |
| 18. Providing pain relief |  |  |  |
| 19. Paying attention to safety in providing care and interventions |  |  |  |
| 20. Providing accurate information about progress of labor to companions |  |  |  |
| 21. Attendance of unnecessary person during performing procedure |  |  |  |
| 22. Physical violence in the case of non-cooperation |  |  |  |
| 23. Shouting at the laboring woman in case of non-cooperation |  |  |  |

| **Practice section** | | | | | |
| --- | --- | --- | --- | --- | --- |
| **Items** | **Always** | **Often** | **Sometimes** | **Rarely** | **Never** |
| 1. I welcome laboring woman warmly. |  |  |  |  |  |
| 2. I introduce myself to the laboring woman. |  |  |  |  |  |
| 3. I show the laboring woman around the labor unit. |  |  |  |  |  |
| 4. I establish friendly and appropriate relationship with the laboring woman. |  |  |  |  |  |
| 5. I support laboring woman by encouraging and calming touch. |  |  |  |  |  |
| 6. I use the name preferred by a laboring woman. |  |  |  |  |  |
| 7. I am continuously or timely available beside. |  |  |  |  |  |
| 8. I provide laboring woman with correct and clear information about the care, interventions and progress of labor. |  |  |  |  |  |
| 9. I build friendly relationship in a way that she feels comfortable to ask her questions. |  |  |  |  |  |
| 10. I provide a comfortable environment for laboring woman. |  |  |  |  |  |
| 11. I support laboring woman to be in her desired birthing position. |  |  |  |  |  |
| 12. I keep medical records and the results of examinations and consultations confidential. |  |  |  |  |  |
| 13. I cover the laboring woman’s body during examinations, using sheets. |  |  |  |  |  |
| 14. I perform all interventions with laboring woman’s informed consent. |  |  |  |  |  |
| 15. I provide equal care to all women, regardless of their socio-economic status, ethnicity, etc. |  |  |  |  |  |
| 16. I support laboring woman to take care of herself and her baby. |  |  |  |  |  |
| 17. I provide evidence-based and up-to-date childbirth care. |  |  |  |  |  |
| 18. I pay attention to laboring woman’s safety in providing care and interventions. |  |  |  |  |  |
| 19. I respect beliefs and culture of laboring woman and her companions. |  |  |  |  |  |
| 20. I provide companions with accurate and clear information about progress of labor. |  |  |  |  |  |
| 21. I do not allow the laboring woman to have companion inside the labor unit. |  |  |  |  |  |
| 22. I may beat the laboring woman if she does not cooperate. |  |  |  |  |  |
| 23. I may shout at laboring if she does not cooperate. |  |  |  |  |  |
